# Age period cohort trends in alcohol treatment episodes across Australia from 2003 to 2022

**DOI:** 10.1111/add.70450

**Published:** 2026-04-29

**Authors:** Wing See Yuen, Mia Miller, Nicola Man, Michael Livingston, Agata Chrzanowska, Philip Clare, Jane Akhurst, Louise Tierney, Kristina Da Silva, Parker Blakey, Willow Bryant, Gary Chan, Janni Leung, Amy Peacock

**Affiliations:** ^1^ National Drug and Alcohol Research Centre UNSW Sydney Sydney Australia; ^2^ National Drug Research Institute, enAble Institute, Faculty of Health Sciences Curtin University Perth Australia; ^3^ Prevention Research Collaboration, Sydney School of Public Health The University of Sydney Camperdown Australia; ^4^ Charles Perkins Centre The University of Sydney Camperdown Australia; ^5^ Australian Institute of Health and Welfare Canberra Australia; ^6^ National Centre for Youth Substance Use Research, School of Psychology The University of Queensland Brisbane Australia

**Keywords:** age period cohort, alcohol, alcohol epidemiology, alcohol treatment, descriptive epidemiology, trends

## Abstract

**Aims:**

To measure trends in alcohol treatment episodes in Australia, disaggregated by age, period and birth cohort.

**Design and setting:**

Age, period, cohort modelling with restricted cubic splines, using Australian alcohol treatment administrative data from July 2002 to June 2022.

**Participants:**

1 253 548 closed treatment episodes where alcohol was the primary drug of concern from people aged 10 to 100 years who received treatment for their own substance use in publicly funded specialist alcohol and other drug treatment services.

**Measurements:**

Count of alcohol treatment episodes by age, period, birth cohort and sex.

**Findings:**

Alcohol treatment episode rates increased over time, peaking in 2022 (330.11 per 100 000 population). Age trends first peaked at around 21 years of age [cross‐sectional prevalence = 444.30, 95% confidence interval (CI) = 440.82–447.80; longitudinal prevalence = 462.45, 95% CI = 458.06–466.89], followed by a lifetime peak between 37 and 44 years and declining with older age. Cohorts born from 1974 to 1979 had the highest alcohol treatment episode rates, and the oldest and youngest birth cohorts had the lowest alcohol treatment episode rates. Males were overall 1.8 times as likely as females to have an alcohol treatment episode, but this gap closed with more recent birth cohorts.

**Conclusions:**

Alcohol treatment episode rates increased in Australia between 2003 and 2022, and particularly from 2017. Young to middle‐aged adults and people born in the 1970s were most at risk, alongside a persistent but narrowing gap between males and females.

## INTRODUCTION

Alcohol use is the ninth leading risk factor for death and disability world‐wide, and the leading risk factor for people 15 to 49 years [1, 2]. The most recent Global Burden of Disease Study reports that exposure to alcohol has increased at a ‘concerning’ rate from 2010 to 2019, with years of life lost because of alcohol use increasing substantially, particularly in low and middle income regions [2]. Although many high‐income countries across Europe, North America and Oceania have observed population‐level declines in alcohol consumption [3, 4, 5, 6], emerging evidence suggests increasing or relatively stable proportions of people who drink at risky levels [7, 8, 9] and experience harms attributable to alcohol use [10, 11, 12].

In Australia, population surveys have found less risky behaviour undertaken while under the influence of alcohol (e.g. driving) from 2001 to 2016, with the exception of the 50+ age group, who reported more risky behaviours over time [13]. This coincides with decreases in the prevalence and level of Australian alcohol use across the same period that has been driven by more recent birth cohorts (i.e. people born 1995–1999) [5]. Alcohol‐attributable hospitalisations across Australia also appear to be decreasing in younger people (age 15–34 years), but are increasing in people age 35 years and older [14]. In particular, alcohol‐attributable hospitalisations and deaths have been increasing sharply in people age 65 years and older between 2010 and 2017 [14].

Nevertheless, trends in use and harm do not necessarily translate to similar trends in treatment for alcohol problems. Many of the harms from alcohol, such as chronic diseases, accidents and injuries, can arise from patterns of drinking that fall below the threshold for an alcohol use disorder (AUD) diagnosis [15], and only a small proportion of people with an AUD receive treatment [16, 17]. A global systematic review of studies between 2004 and 2019 found that 9.3% to 22.3% of people with AUDs received treatment, with particularly low rates in low and lower‐middle‐income countries [17]. In Australia, approximately 22% of people with an AUD sought treatment in 2007 [18] and only 3% of people with alcohol dependence received pharmacotherapy between 2009 and 2013 [19]. The only study that has statistically examined trends in alcohol treatment episodes in Australia since the 21st century reported that the rate of treatment episodes increased among younger age groups (14–35 years of age) between 2002 and 2011, then declined slightly between 2011 and 2013 [20]. In contrast, treatment episode rates in older age groups were relatively stable with slight increases between 2002 and 2013 [20]. The same study also reported that treatment episode rates increased sharply between 2002 and 2013 for cohorts born during or after the 1980s, but not for earlier birth cohorts [20]. However, it is unclear whether it is the effect of age, cohort or both that are driving these trends. For instance, the reported rates in birth cohorts may be because of the effects of ageing rather than being cohort‐specific, as people born in the 1990s were below the Australian legal age of alcohol purchase and consumption at a licensed venue for at least half of the data collection period in this study [20].

Treatment seeking and utilisation for alcohol problems also appear to vary between males and females. One study in the United States reported that women were much less likely to use alcohol treatment services compared to men, although some factors that influence treatment utilisation (e.g. court mandates) were not assessed [21]. Conversely, data from a nationally representative Australian survey conducted in 2007 showed that 20% of males and 26% of females with symptoms consistent with an AUD in the year prior had accessed health services for mental health problems during that same period [18]. Since the early 2000s, there has been a decline in the rates of alcohol consumption and risky drinking among males, whereas rates among females have been stable or increasing depending on age [22, 23]. Therefore, there may also be similar male–female variations by age and birth cohort in treatment episodes.

### Aims

This study aimed to examine trends in rates of alcohol treatment episodes in Australia, disentangling the extent to which variation over time was driven by age (changes across the life course), period (population‐level temporal changes) and cohort (differences between birth cohorts) and assessing whether these effects differed between males and females.

## METHODS

### Participants and data

We used the Alcohol and Other Drug Treatment Services National Minimum Data Set (AODTS‐NMDS), a collection of de‐identified administrative data on alcohol and other drug treatment episodes in Australia from people age 10 to 100 years between 1 July 2002 and 30 June 2022. The AODTS‐NMDS comprises data from publicly funded government and non‐government agencies providing specialist alcohol and other drug treatment services. Further information about the scope of the dataset can be found in the web report *Alcohol and other drug treatment services in Australia: Annual report* [24]. The AODTS‐NMDS captures closed episodes where treatment is completed, when there has been no contact between the client and provider for 3 months, where there is a change in the main treatment type, principal drug of concern or delivery setting or where treatment has ceased. Consequently, the same client can receive multiple treatment episodes.

### Measures

Episodes where alcohol was recorded as the client's own principal drug of concern were considered as alcohol treatment episodes (restricted for the purposes of this study to beverage alcohols, i.e. codes 2100, 2101 of the Australian Standard Classification of Drugs of Concern). Age at the commencement of treatment and year of treatment commencement were included as continuous integer variables. Year of birth was calculated from clients' age and date at commencement of treatment. Sex is recorded administratively in the dataset as ‘male’, ‘female’, ‘other’ or ‘not stated/inadequately described’. We included sex as a categorical variable to examine differences in trends between males and females, with episodes where sex was identified as ‘other’ or ‘not stated/inadequately described’ not included because of concerns about data reliability.

### Analyses

Our analysis plan was pre‐registered (https://osf.io/c3kuy) and reporting follows Strengthening the Reporting of Observational Studies in Epidemiology (STROBE) guidelines (Appendix A). Crude rates of treatment episodes per 100 000 population for males and females were calculated using estimated resident population at 30 June of each year as provided by the Australian Bureau of Statistics [25]. These crude rates were used to examine whether an age, period, cohort (APC) model is justified, where an age‐period (AP) model is supported if age‐specific rates are proportional between periods and an age‐cohort (AC) model is supported if age‐specific rates are proportional between cohorts. To facilitate visual interpretation of age, period and cohort patterns, crude rates were also presented as ‘hexamaps’ [26] for males, females and persons.

#### APC modelling

Our primary analysis was an APC model to examine trends in alcohol treatment episode rates, conducted using Stata 16.1 [27]. Because the APC components are linearly dependent (e.g. period – age = cohort), there is an ‘identification problem’ where modelling each of these effects results in the exclusion of one component because of over parameterization. There is no best solution for this problem, with different modelling approaches potentially leading to different results [28]. We used Rutherford's APC approach, which models age, period and cohort effects using restricted cubic spline functions in a Poisson regression model with log (population) as the offset, implemented using the *apcfit* Stata package [29]. Equally spaced knots in the restricted cubic spline function determine time points where piecewise cubic polynomials join to allow for smoothness in each of APC trends and a higher number of knots allows for more wiggling in the trends. This approach uses model constraints to address the identification problem. As age is a major risk factor in many types of alcohol‐related behaviours, linear temporal changes (‘drifts’) were attributed to either the cohort or the period functions. The age function is presented in two forms:

1. Age‐period‐cohort (AP‐C) (i.e. cross‐sectional age‐specific rates adjusting for cohort effects) where drift was included in the period function. This age function shows how people of different ages in a particular year differ. The period function was set to zero for 2013, the median year of data. Period effects were presented as prevalence ratios (PR) with respect to the median year, and the cohort function was represented as residuals in relation to estimates for age and period effects.
2. Age‐cohort‐period (AC‐P) (i.e. longitudinal age‐specific rates adjusting for period effects) where drift was included in the cohort function. This age function shows how people in a particular birth cohort change as they become older. The cohort function was set to zero for 1974, the median birth cohort. Cohort effects were presented as PR with respect to the median cohort, and the period function was represented as residuals in relation to estimates for age and cohort effects.

This approach allows for clear graphical depiction of non‐linear APC trends and accommodates the inclusion of covariates, which we used in the current study to compare trends between males and females [29]. The number of knots in the cubic spline function was chosen based on a scree plot of the fit statistics [i.e. Akaike information criterion (AIC) and Bayesian information criterion (BIC), against the number of knots for the full APC model]. The model with number of knots that showed minimal decrease in AIC and BIC with increasing number of knots in the scree plot (i.e. increasing number of knots showed no substantial improvement in model fit) was chosen for the main analysis.

#### Male–female APC interaction effects

To examine male–female effects, we fitted interaction models with a reduced number of splines and calculated time‐dependent PR [30]. With females as the reference, PRs equal to 1.0 indicate no detectable differences between male and female prevalence of treatment episodes, whereas PRs greater than 1.0 indicate that males have higher prevalence compared to females and lower prevalence for PRs less than 1.0. Since the identifiability problem is reintroduced because of the interaction term, one of the APC terms needs to be excluded. As the convergence in male and female alcohol consumption appears to largely be dependent on age and cohort rather than period [22, 23], we fitted models for:

1. Age interaction adjusting for birth cohort, where the period effect is assumed to be similar between males and females; and
2. Cohort interaction adjusting for age, where the period effect is similarly assumed to be comparable across males and females.

To confirm that alcohol treatment prevalence did not substantially differ between males and females by period, separate male and female APC models were estimated and the period effect was compared.

#### Sensitivity analyses

Given that the AODTS‐NMDS includes episodes that are categorised only as ‘assessment’ (i.e. no treatment was provided), we conducted a sensitivity analysis excluding assessment‐only episodes using the same APC method as the primary analyses to examine whether trends were substantially impacted by this exclusion.

Additionally, to examine whether the trends vary depending on the APC method used for the model, we repeated the primary APC analysis using a generalised linear model with weighted least squares (WLS) approach and Poisson distribution assumption that has previously been used in substance‐related epidemiology research [31, 32]. This analysis was conducted using R [33] with a function written by Rosenberg *et al*. [32]. Similar to the primary analyses, longitudinal and cross‐sectional age‐specific rates are estimated, as are period‐specific and cohort‐specific relative risks. Net drifts and local drifts are reported, where net drift is the overall annual percentage change in treatment episode rates, and local drifts are annual percentage changes at each age.

## RESULTS

### Crude rates of alcohol treatment episodes

Note that the years referred to in this study are Australian fiscal years (i.e. ‘2003’ refers to 1 July 2002 to 30 June 2003). The annual number of alcohol treatment episodes and crude rates per 100 000 population are shown in Table 1. Treatment episode rates were higher in 2022 than 2003 for males, females and for all persons. Treatment episode rates peaked at approximately 2008 to 2010 for males and all persons, whereas rates were highest in 2022 for females. Rates of alcohol treatment episodes increased from 2003, declined between 2010 and 2017, and subsequently increased among both males and females.

**TABLE 1:** Annual alcohol treatment episodes by males, females and persons.

Year	Treatment episodes *n* (per 100 000 population)			Treatment episodes, excluding assessment‐only *n* (per 100 000 population)		
	Males	Females	Persons	Males	Females	Persons
2003	32 015 (378.68)	13 697 (158.03)	45 712 (266.98)	26 486 (313.28)	12 460 (143.75)	38 946 (227.46)
2004	33 357 (389.46)	14 534 (165.67)	47 891 (276.22)	26 943 (314.57)	12 819 (146.12)	39 762 (229.33)
2005	34 714 (399.65)	14 906 (167.63)	49 620 (282.28)	28 734 (330.8)	13 435 (151.09)	42 169 (239.89)
2006	35 600 (403.81)	16 383 (181.71)	51 983 (291.51)	28 040 (318.06)	14 228 (157.81)	42 268 (237.03)
2007	38 411 (427.40)	17 324 (188.79)	55 735 (306.85)	30 499 (339.36)	14 851 (161.84)	45 350 (249.68)
2008	44 657 (486.56)	19 799 (211.68)	64 456 (347.82)	36 514 (397.84)	17 405 (186.08)	53 919 (290.96)
2009	43 793 (466.93)	18 927 (198.37)	62 720 (331.50)	35 303 (376.41)	16 576 (173.73)	51 879 (274.20)
2010	46 222 (485.34)	20 679 (213.32)	66 901 (348.12)	37 817 (397.09)	18 381 (189.61)	56 198 (292.43)
2011	46 417 (480.63)	21 131 (214.82)	67 548 (346.50)	38 596 (399.65)	18 800 (191.12)	57 396 (294.42)
2012	44 011 (448.33)	20 628 (206.22)	64 639 (326.14)	36 876 (375.65)	18 210 (182.05)	55 086 (277.94)
2013	42 089 (422.00)	20 074 (197.36)	62 163 (308.58)	34 408 (344.99)	17 477 (171.82)	51 885 (257.56)
2014	45 537 (450.52)	23 272 (225.29)	68 809 (336.68)	37 768 (373.66)	20 389 (197.38)	58 157 (284.56)
2015	39 817 (388.76)	20 695 (197.37)	60 512 (291.95)	32 616 (318.45)	17 606 (167.91)	50 222 (242.30)
2016	39 723 (382.29)	20 955 (196.59)	60 678 (288.26)	32 799 (315.65)	17 814 (167.13)	50 613 (240.44)
2017	38 530 (364.19)	21 535 (198.47)	60 065 (280.28)	31 859 (301.13)	18 239 (168.09)	50 098 (233.77)
2018	44 858 (416.80)	24 254 (219.84)	69 112 (317.10)	37 237 (345.99)	20 637 (187.05)	57 874 (265.54)
2019	45 832 (418.65)	26 019 (231.97)	71 851 (324.17)	37 218 (339.96)	21 930 (195.51)	59 148 (266.86)
2020	45 291 (407.78)	25 892 (227.36)	71 183 (316.44)	36 507 (328.69)	22 004 (193.22)	58 511 (260.11)
2021	47 779 (428.81)	28 635 (250.79)	76 414 (338.71)	38 455 (345.13)	24 237 (212.27)	62 692 (277.89)
2022	46 469 (411.13)	29 087 (251.07)	75 556 (330.11)	36 737 (325.03)	23 961 (206.83)	60 698 (265.20)

*Note*: Year refers to Australian fiscal years (i.e. ‘2003’ is the period of 1 July 2002 to 30 June 2003.

**FIGURE 1:**
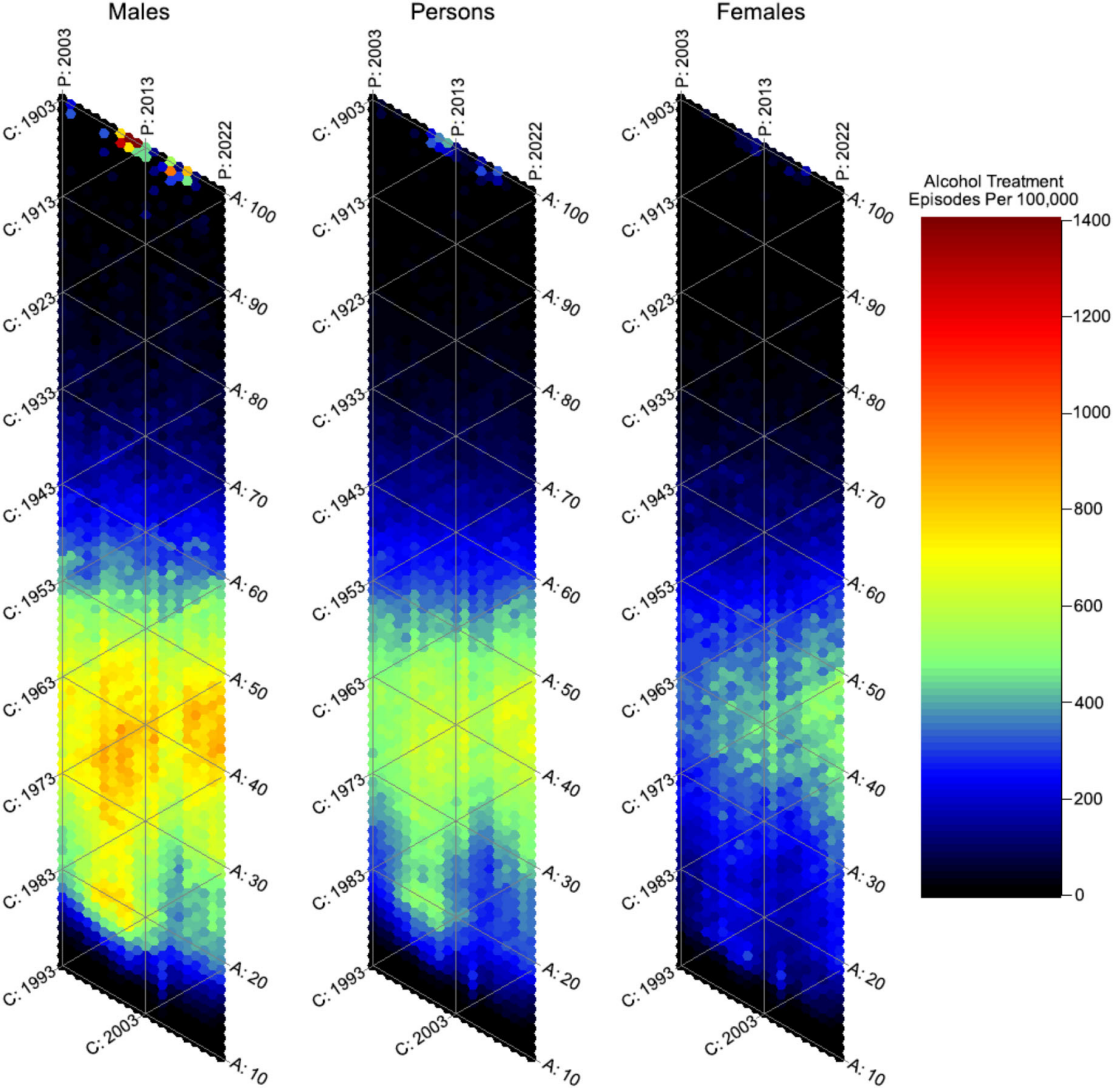
Age, period, cohort hexamaps of alcohol treatment episodes per 100 000 population by males (left), persons (centre) and females (right). A, age; P, period; C, cohort.

Hexamaps indicated APC patterns (Figure 1). Higher rates of alcohol treatment episodes were observed in the 18 to 22 age group among male birth cohorts born between 1985 and 1993 or in the period between 2005 and 2012, and more generally across the male birth cohorts from approximately the ages of 30 to 50. It was also higher among females 35 to 50 years old in the birth cohorts born around 1970 or later. Higher rates of alcohol treatment episodes were observed among males born approximately between 1963 and 1985 and females born around 1970 onward. Age‐specific rates by period and period specific rates by age group were roughly parallel, showing evidence of age‐cohort effects (Appendix B1). Similarly, age‐specific rates by birth cohort and cohort specific rates by age group showed evidence of age‐cohort effects (Appendix B2).

### APC models

**FIGURE 2:**
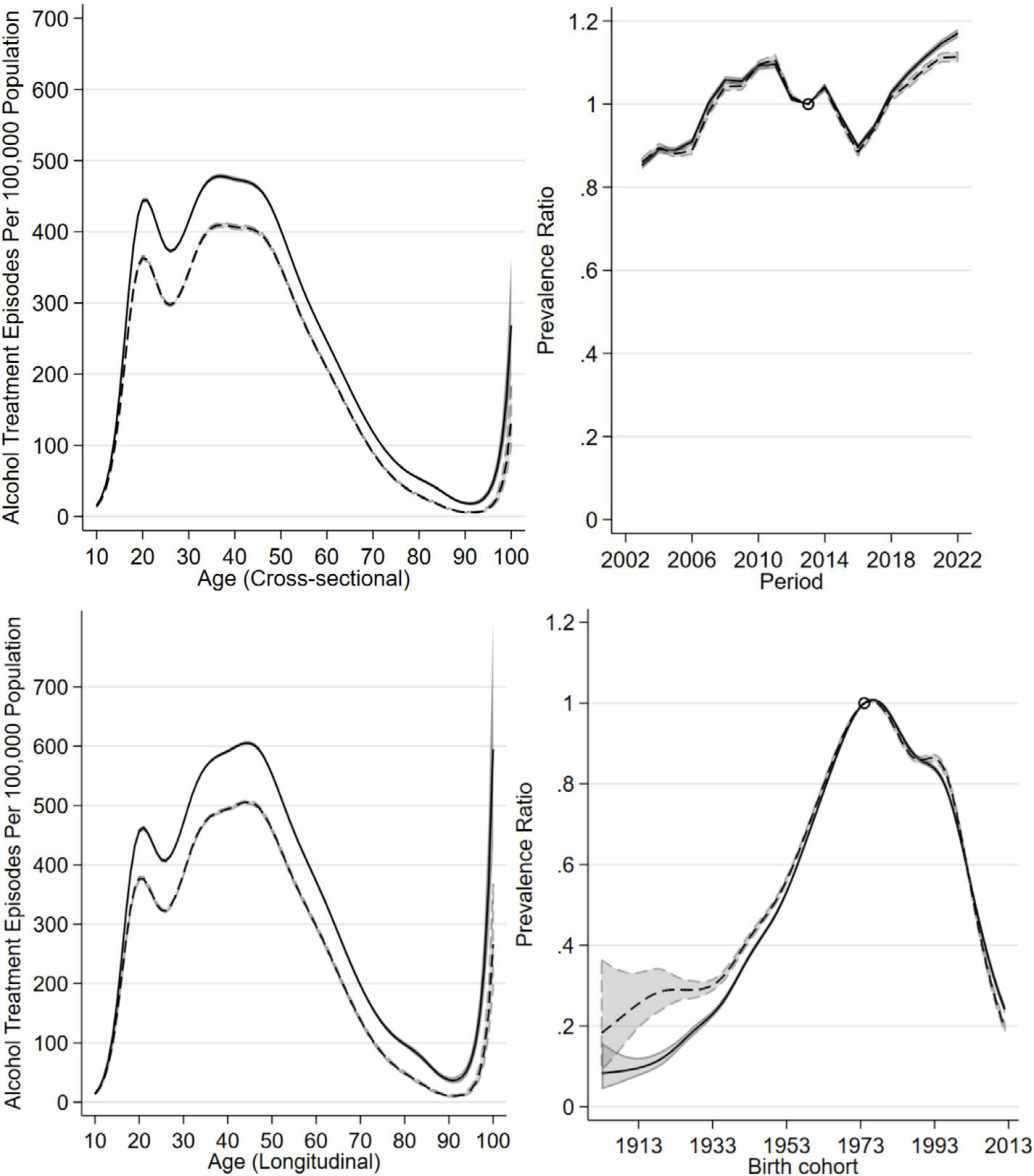
Estimated effects with 95% CI from age‐period‐cohort (APC) models using age‐period‐cohort (AP‐C; top) and age‐cohort‐period (AC‐P; bottom) functions for alcohol treatment episodes per 100 000 people with 11 internal knots for age, period and birth cohort. Cross‐sectional AP‐C age effects for the reference period of 2013 are shown on the top left. AP‐C period effects are shown on the top right. Longitudinal AC‐P age effects for the reference birth cohort of 1974 are shown on the bottom left. AC‐P cohort effects are shown in the bottom right. Solid lines are estimates from the primary models, dashed lines are estimates from the sensitivity models where assessment‐only treatment episodes are excluded.

Details of our model selection process can be found in Appendix C1. To describe the non‐linear effects in our primary APC models, we used 11 equally spaced internal knots for each of age, period and cohort. Table 2 shows the fit statistics for this model, with the full APC model having improved fit over the AP and AC models (see Appendix D for fit statistics using other counts of internal knots and Appendix E for plotted AIC and BIC values). The AC model had better fit than the AP model at all knot counts, indicating that cohort had a more pronounced effect than period. Fitted values for the two forms of APC functions, AP‐C and AC‐P, are shown in Figure 2 and residuals are shown in Appendix F.

**TABLE 2:** Fit statistics for primary age‐period‐cohort‐model.

Model	AIC	BIC	Log‐likelihood	d.f.	Deviance	*P*
APC	15.81	3087.84	−14354.90	1784	–	–
AP	26.68	22809.58	−24257.06	1795	36283.91	<0.001
AC	19.02	8859.89	−17282.21	1795	22334.23	<0.001

*Note*: Using 11 equally spaced internal knots for each of age, period and cohort.

Abbreviations: AC, age‐cohort; AIC, Akaike information criterion, smaller values indicate better model fit; APC, age‐period‐cohort; AP, age‐period; BIC, Bayesian information criterion, smaller values indicate better model fit.

#### Age trends

Both the cross‐sectional (AP‐C; Figure 2 top left solid line) and longitudinal (AC‐P; Figure 2 bottom left solid line) age trends showed that the prevalence of alcohol treatment episodes increased from age 10 years [cross‐sectional prevalence = 15.52, 95% confidence interval (CI) = 14.99–16.07; longitudinal prevalence = 14.54, 95% CI = 14.03–15.06] to an initial peak at approximately age 21 years (cross‐sectional = 444.30, 95% CI = 440.82–447.80; longitudinal = 462.45, 95% CI = 458.06–466.89), followed by a trough at approximately age 26 years (cross‐sectional = 372.64, 95% CI = 369.77–375.54; longitudinal = 406.89, 95% CI = 403.47–410.33). Cross‐sectional age trends showed a peak at approximately age 37 years (478.08, 95% CI = 474.73–481.45), whereas longitudinal age trends peaked at approximately age 44 years (605.40, 95% CI = 602.24–608.58), and steadily declined thereafter. Although the prevalence of treatment episodes appeared to increase sharply from age 93 onward, the CIs for these estimates were very wide and should, therefore, be interpreted with caution (e.g. cross‐sectional prevalence at age 100 = 268.50, 95% CI = 198.50–363.17).

#### Period trends

All effects reported here are relative to 2013 as the reference. As shown in Figure 2 (top right solid line), the rate of alcohol treatment episodes increased steadily from 2003 with an initial peak at approximately 2011 (PR = 1.10, 95% CI = 1.09–1.11). Alcohol treatment episode rates fluctuated between 2011 and 2016, with a trough in 2016 (PR = 0.90, 95% CI = 0.89–0.91). From 2017 onward, the rate of treatment episodes steadily increased, with the most recent period having the highest rate of alcohol treatment episodes (2022 PR = 1.17, 95% CI = 1.16–1.18).

#### Cohort trends

Relative to the 1974 birth cohort, most other cohorts had significantly lower rates of alcohol treatment episodes (Figure 2, bottom right solid line). The 1975 to 1979 birth cohorts were the exception, with slightly higher or similar rates to the 1974 cohort. In general, PRs decreased the further a birth cohort was from the 1974 to 1979 cohorts. CIs widened with older birth cohorts from approximately the 1933 cohort, reflecting less precise estimates likely because of the relatively low resident population in these cohorts.

### Male–female interaction models

**FIGURE 3:**
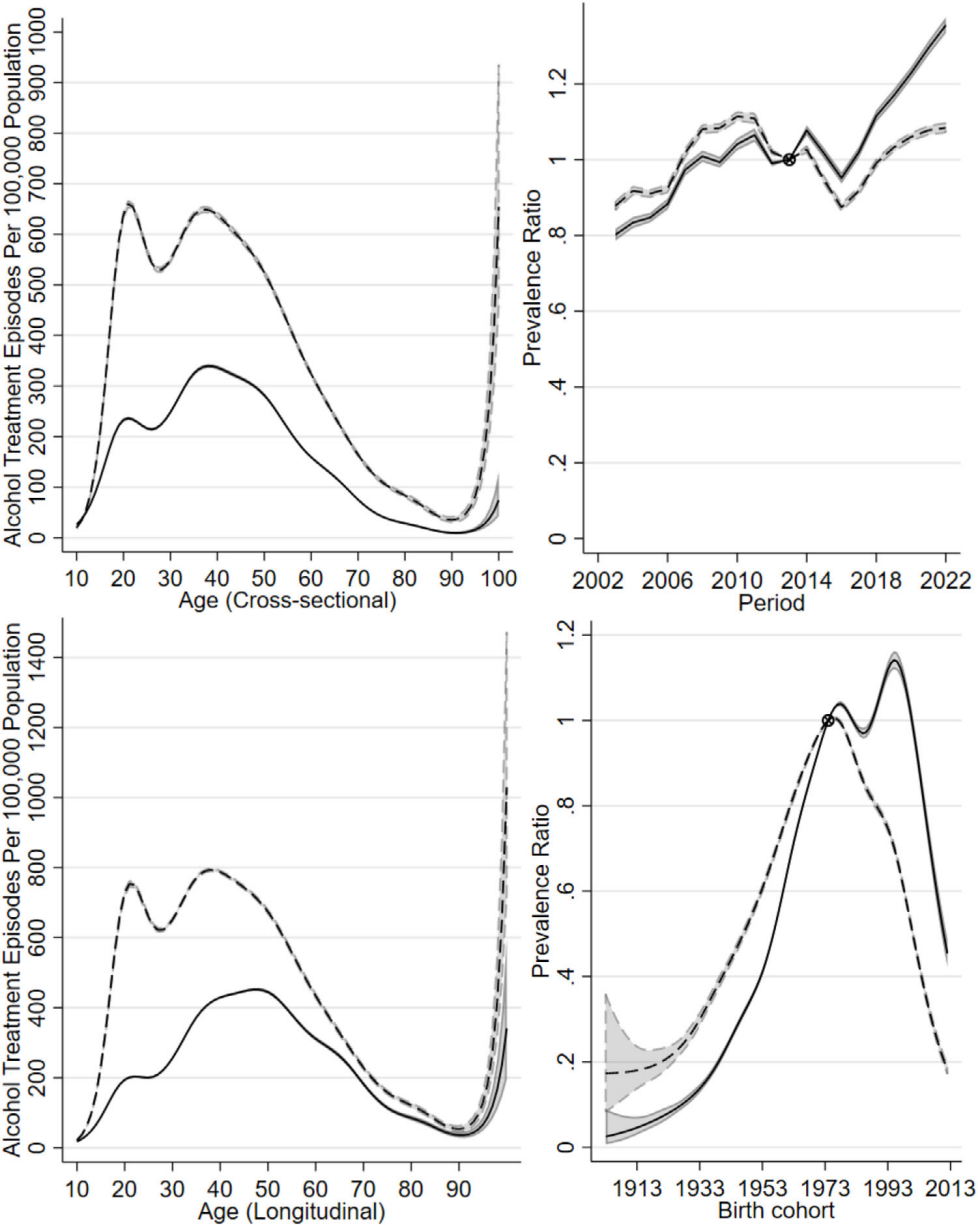
Estimated effects with 95% CI from separate male (dashed lines) and female (solid lines) age‐period‐cohort (APC) models using age‐period‐cohort (AP‐C; top) and age‐cohort‐period (AC‐P; bottom) functions for alcohol treatment episodes per 100 000 people with 11 internal knots for age, period and birth cohort. Cross‐sectional AP‐C age effects for the reference period of 2013 are shown on the top left. AP‐C period effects are shown on the top right. Longitudinal AC‐P age effects for the reference birth cohort of 1974 are shown on the bottom left. AC‐P cohort effects are shown in the bottom right.

**FIGURE 4:**
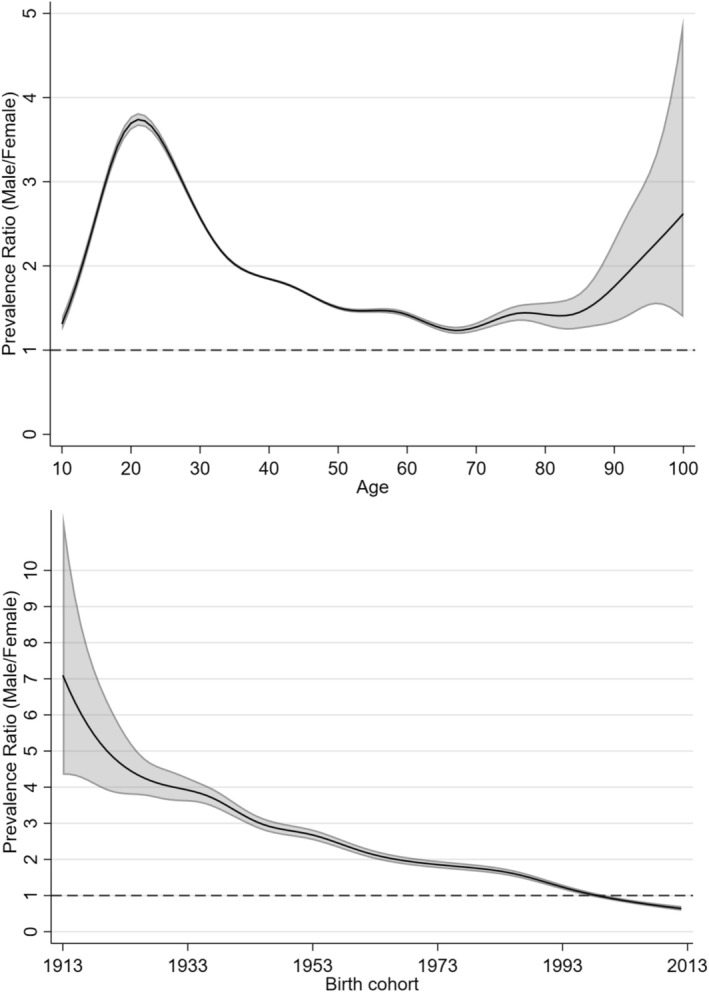
Estimated time‐dependent prevalence ratios with 95% CI for males with females as the reference in reduced splines model with 10 knots for age, period and cohort. Age effects adjusting for cohort effects are shown on the top panel. Cohort effects adjusting for age effects are shown on the bottom panel. Effects above the dashed line (prevalence ratio = 1.0) indicate that males have a higher prevalence of alcohol treatment episodes than females, whereas effects below the dashed line indicate females have a higher prevalence than males.

The assumption for the male–female interaction models (i.e. that the period effect does not substantially differ between males and females) was generally supported as separate APC models for males and females showed similar fluctuations in the period effect, although from 2018 onward the PR for males appeared to increase at a steeper rate compared to females (Figure 3, top right). To describe time‐dependent non‐linear effects in our reduced‐spline male–female interaction models, we used 10 equally spaced internal knots for the age and cohort components (see Appendix C2 for model selection details and Appendix G for fit statistics). Time‐dependent PRs for males versus females for the age and cohort effects are shown in Figure 4. A male–female interaction effect was present, with males being more likely to have an alcohol treatment episode compared to females (PR = 1.84, 95% CI = 1.74–1.94). The age interaction effect size increased sharply between age 10 (PR = 1.31, 95% CI = 1.23–1.40) and age 21 years (PR = 3.74, 95% CI = 3.66–3.82), then steadily declined but remained above 1.00 at all ages. Although there appeared to be an increase from age 85 years, the estimates at these oldest ages were less precise. The cohort interaction effect size was highest in the earliest birth cohorts (1903 PR = 13.86, 95% CI = 3.35–57.37), although estimates at these birth cohorts were less precise. The cohort interaction effect size steadily declined, with the PR decreasing below 1.00 for the first time with the 2001 cohort (PR = 0.92, 95% CI = 0.86–0.97) and continued to decline up to the most recent cohort (2012 PR = 0.64, 95% CI = 0.58–0.71).

### Sensitivity analyses

Results of the sensitivity analyses aligned with the primary analyses. There were some differences in the effect sizes, but the effects were broadly similar (e.g. the WLS approach showed a cross‐sectional lifetime peak at age 20 years rather than 37 years, although the trend was overall very similar to the primary analysis). For further details of the sensitivity analyses results, see Appendix H.

## DISCUSSION

Our study found that publicly funded alcohol treatment episode rates in Australia increased slightly over time between 2003 and 2022, with large differences by age and birth cohort. Alcohol treatment episode rates showed a lifetime peak in individuals aged between their mid‐30s and mid‐40s. Cohorts born in the mid‐late 1970s had the highest prevalence of alcohol treatment episodes, with the oldest and youngest birth cohorts having the lowest prevalence. Males were overall 1.8 times as likely to have alcohol treatment episodes compared to females. This disparity was largest at approximately 20 to 25 years of age and smallest with the most recent birth cohorts.

Given the small effect size and substantial fluctuations, the overall increase in alcohol treatment episodes observed between 2003 and 2011 may not be of particular clinical importance as an indicator for trends in chronic alcohol‐related harm. However, trends from 2017 onward suggest real increases in treatment rates among the Australian population. These findings contrast with declining treatment episodes in the United States over the past two decades [34], linked to greater barriers to treatment‐seeking (e.g. costs, service availability) [16, 35], as well as more recent coronavirus disease 2019 (COVID‐19) disruptions in treatment admissions [36]. Individual‐level data on the number of unique clients accessing treatment were not available for inclusion in our analysis, however, aggregate data published elsewhere shows an increase in the number of unique individuals accessing treatment in Australia, from 42 027 in 2017to 201818 to 50 106 in 2022 to 2023, equating to an increase in the rate of clients per 100 000 from 195 to 216 [37]. Concurrently, the average number of treatment episodes per client remained relatively stable over time, at 1.8 episodes per client [37]. This rise in treatment engagement may be a direct signal of harm and service need [37], as the increase aligns with rises in other indicators of alcohol‐related harm, such as alcohol‐induced deaths [38] and hospitalisations for alcohol‐related liver disease [39]. It could also reflect a narrowing of the treatment gap because of improved availability and range of services, reduced stigma or greater awareness among consumers of alcohol‐related problems and how to seek help. For example, there was an increase in alcohol and other drug treatment agencies reporting to AODTS‐NMDS from 2016 to 2017 to 2018 to 2019, attributed to increased Commonwealth service funding through various initiatives [24]. Expansion of online treatment modalities in response to COVID‐19 restrictions may have increased the accessibility of treatment [40], and data shows that the median treatment delay for AUD in Australia has decreased substantially over time [41]. Robust, population‐level estimates of treatment need and coverage are critical to further advance understanding of these potential drivers of increased utilisation.

The initial peak in treatment episodes occurred at approximately 21 years of age. Epidemiological evidence from Australia shows that risky alcohol consumption (i.e. in excess of Australian alcohol intake guidelines) and engagement in alcohol‐related risk behaviours also peaks at this age [42, 43]. These behaviours may prompt young people to seek treatment directly or place them in contact with health, social or justice services, which may offer voluntary or mandated treatment referral pathways. Later peaks in middle age and older reflect the long lags between onset of AUDs and treatment access [44], as well as increases in chronic heavy drinking and associated health impact that typically occur during adulthood. The peak in alcohol treatment episode rates with the late 1970s birth cohorts roughly corresponds to population survey data in Australia, which shows that cohorts born in the 1950s to 1970s generally have higher prevalence of any alcohol use and consume greater volumes of alcohol compared to other birth cohorts [5]. Similarly, the substantial and steady decline in alcohol treatment episode rates with more recent birth cohorts is commensurate with reports of declining alcohol use, risky alcohol use and alcohol‐related harm among young people in Australia [4, 5, 14, 43, 45] and other similar countries such as the United Kingdom [7]. Our findings are also reflective of cohort‐related changes in attitudes and norms toward alcohol in Australia, namely that more people in the most recent birth cohorts (i.e. 1990s onward) tend to disapprove of regular alcohol use and consider alcohol to be harmful compared to older birth cohorts [46]. Although these are promising trends for younger cohorts, alcohol use problems remain an important public health issue in Australia, especially among those born in the mid‐20th century.

Aligning with well‐established trends in alcohol consumption [43, 47], alcohol treatment episode rates were overall much higher among males compared to females. However, this gap closed with more recent birth cohorts, which mirrors findings on the closing gender gap in alcohol consumption [22, 45, 47] and harms [14, 42] between males and females. It is also possible that treatment rates among females are underestimated in our study, because they are more likely to access treatment through mental health, primary care or online services [48, 49], which are not captured in our dataset [24]. Recent estimates suggest that people receiving treatment in these other settings represent approximately one‐third of all clients receiving alcohol treatment per year in Australia, however, sex‐disaggregated data are not currently available [50]. Although our analyses cannot show the exact drivers of the male–female convergence in alcohol treatment episode rates, risky alcohol use in Australia has been declining at a faster rate among males than females [43], as are alcohol‐related harms [14, 43]. Therefore, the closing gap in alcohol treatment observed in our study is likely to be at least partially attributable to the trends observed in alcohol consumption, although further research is needed to specifically identify the underlying causes of this shift. These changes also highlight the importance of tailoring public health interventions to address sex differences in risk factors and drivers of alcohol consumption, particularly as policies aimed at reducing alcohol‐related harms across the population can unintentionally have differential effects by sex [51].

### Limitations

There are several important limitations to consider in the context of our findings. As acknowledged above, the AODTS‐NMDS only contains data from publicly funded agencies with specialist treatment services [24], meaning that overall alcohol treatment episode rates in Australia are underestimated in this analysis. Future data linkage studies are needed to determine access to other treatment services by gender, given women are more likely to use services offered outside of publicly funded agencies. The dataset also does not include person identifiers, as such, we are unable to account for repeat episodes within the same person (i.e. our results should be interpreted as trends at the episode level rather than the person level). There have also been changes in the funding and reporting systems for alcohol and other drug treatment across different states and territories that should be caveated, but these most likely impact the comparability of episodes across these jurisdictions and not the overall reliability of the results for the full sample [24]. However, other structural changes occurring across the country, such as reductions in the total population size during the COVID‐19 pandemic because of border closures, could have resulted in artificially inflated rates in these periods [52]. These limitations only impact on the period effects estimated and should not influence age‐ or cohort‐ patterns. Estimates were less precise for the oldest and youngest cohorts because of low population size and low prevalence, respectively. Finally, one of the main criticisms of APC modelling is that the effects estimated can vary depending on the method used to account for the inherent relationship between age, period and cohort. However, sensitivity analyses using an alternative approach produced similar results, supporting the robustness of the findings.

## CONCLUSION

Publicly funded alcohol treatment episode rates in Australia broadly increased between 2003 and 2022 (particularly from 2017), with at‐risk groups being adults 20 to 50 years old and people who were born around the 1970s. Alcohol treatment rates remains much higher among males than females, although this disparity is narrowing with more recent birth cohorts. As our findings align with existing evidence around alcohol use and harms, these trends might be reflective of broader societal changes related to alcohol rather than being specific to treatment service utilisation. However, robust, population‐level measures of treatment need and coverage are required. Future research should investigate the factors contributing to age‐related differences and the male–female convergence in alcohol treatment episode rates. In doing so, policymakers and healthcare professionals can develop more effective and targeted interventions to reduce alcohol‐related harms and promote more equitable health outcomes across life stages and demographics.

## AUTHOR CONTRIBUTIONS

**Wing See Yuen:** Conceptualization (equal); data curation (equal); formal analysis (equal); writing—original draft (lead); writing—review and editing (equal). **Mia Miller:** Writing—review and editing (equal). **Nicola Man:** Data curation (equal); formal analysis (equal); writing—review and editing (equal). **Michael Livingston:** Conceptualization (equal); data curation (equal); formal analysis (equal); writing—review and editing (equal). **Agata Chrzanowska:** Data curation (equal); writing—review and editing (equal). **Philip Clare:** Formal analysis (equal); writing—review and editing (equal). **Jane Akhurst:** Data curation (equal); writing—review and editing (equal). **Louise Tierney:** Data curation (equal); writing—review and editing (equal). **Kristina Da Silva:** Data curation (equal); writing—review and editing (equal). **Parker Blakey:** Data curation (equal); writing—review and editing (equal). **Willow Bryant:** Data curation (equal); writing—review and editing (equal). **Gary Chan:** Writing—review and editing (equal). **Janni Leung:** Writing—review and editing (equal). **Amy Peacock:** Conceptualization (equal); writing—review and editing (equal).

## DECLARATION OF INTERESTS

M.L. has been a board member of the Alcohol and Drug Foundation, an Australian non‐governmental organization focused on reducing harm from alcohol and other drugs, since 2023. The foundation had no role in study design, conduct or reporting. All other authors have no conflicts of interest to declare.

## Supporting information


**Appendix A.** STROBE Checklist.
**Appendix B.** Age‐period and age‐cohort plots of crude rates.
**Appendix B1.** Age‐specific alcohol treatment episodes per 100,000 population by period (top) and period‐specific alcohol treatment episodes per 100,000 population by age group (bottom).
**Appendix B2.** Age‐specific alcohol treatment episodes per 100,000 population by birth cohort (top) and cohort‐specific alcohol treatment episodes per 100,000 population by age group (bottom).
**Appendix C.** Model selection details.
**Appendix C1.** APC model.
**Appendix C2.** Male‐female APC interaction model.
**Appendix D.** Fit statistics for primary age, period, and cohort models.
**Appendix E.** Plotted AIC (top) and BIC (bottom) values for primary age, period, and cohort models.
**Appendix F.** Residuals from APC models using age‐period‐cohort (left) and age‐cohort‐period (right) functions for alcohol treatment episodes per 100,000 people.
**Appendix G.** Fit statistics for male‐female interaction models.
**Appendix H.** Sensitivity analyses results.
**Appendix H1.** Excluding assessment‐only episodes.
**Appendix H2.** Weighted least squares approach.
**Appendix I.** Fit statistics for age, period, and cohort models excluding assessment‐only episodes.
**Appendix J.** Plotted AIC (top) and BIC (bottom) values for age, period, and cohort models excluding assessment‐only episodes.
**Appendix K.** Weighted least squares APC Wald Tests for alcohol treatment episodes per 100,000 people.
**Appendix L.** Estimated drifts with 95% confidence intervals from weighted least squares APC models for alcohol treatment episodes per 100,000 people.
**Appendix M.** Estimated effects with 95% confidence intervals from weighted least squares APC models for alcohol treatment episodes per 100,000 people.

## Data Availability

The data for this study are publicly available from the Australian Institute of Health and Welfare on request.
