# Supplementary material for: Age period cohort trends in alcohol treatment episodes across Australia from 2003 to 2022
Source: Addiction. 2026 Apr 29;121(8):2154–65. doi: 10.1111/add.70450 (PMC13357926; doi:10.1111/add.70450)
Supplement: Supplementary file 1 — Appendix A. STROBE Checklist. Appendix B. Age‐period and age‐cohort plots of crude rates. Appendix B1. Age‐specific alcohol treatment episodes per 100,000 population by period (top) and period‐specific alcohol treatment episodes per 100,000 population by age group (bottom). Appendix B2. Age‐specific alcohol treatment episodes per 100,000 population by birth cohort (top) and cohort‐specific alcohol treatment episodes per 100,000 population by age group (bottom). Appendix C. Model selection details. Appendix C1. APC model. Appendix C2. Male‐female APC interaction model. Appendix D. Fit statistics for primary age, period, and cohort models. Appendix E. Plotted AIC (top) and BIC (bottom) values for primary age, period, and cohort models. Appendix F. Residuals from APC models using age‐period‐cohort (left) and age‐cohort‐period (right) functions for alcohol treatment episodes per 100,000 people. Appendix G. Fit statistics for male‐female interaction models. Appendix H. Sensitivity analyses results. Appendix H1. Excluding assessment‐only episodes. Appendix H2. Weighted least squares approach. Appendix I. Fit statistics for age, period, and cohort models excluding assessment‐only episodes. Appendix J. Plotted AIC (top) and BIC (bottom) values for age, period, and cohort models excluding assessment‐only episodes. Appendix K. Weighted least squares APC Wald Tests for alcohol treatment episodes per 100,000 people. Appendix L. Estimated drifts with 95% confidence intervals from weighted least squares APC models for alcohol treatment episodes per 100,000 people. Appendix M. Estimated effects with 95% confidence intervals from weighted least squares APC models for alcohol treatment episodes per 100,000 people. [file ADD-121-2154-s001.docx]

**Appendices for Age Period Cohort Trends in Alcohol Treatment Episodes across Australia from 2002 to 2022**

**Table of Contents**

[Appendix A. STROBE Checklist. 2](#_Toc172751864)

[Appendix B. Age-period and age-cohort plots of crude rates. 4](#_Toc172751865)

[Appendix B1. Age-specific alcohol treatment episodes per 100,000 population by period (top) and period-specific alcohol treatment episodes per 100,000 population by age group (bottom). 4](#_Toc172751866)

[Appendix B2. Age-specific alcohol treatment episodes per 100,000 population by birth cohort (top) and cohort-specific alcohol treatment episodes per 100,000 population by age group (bottom). 5](#_Toc172751867)

[Appendix C. Model selection details. 6](#_Toc172751868)

[Appendix C1. APC model 6](#_Toc172751869)

[Appendix C2. Male-female APC interaction model 6](#_Toc172751870)

[Appendix D. Fit statistics for primary age, period, and cohort models. 7](#_Toc172751871)

[Appendix E. Plotted AIC (top) and BIC (bottom) values for primary age, period, and cohort models. 8](#_Toc172751872)

[Appendix F. Residuals from APC models using age-period-cohort (left) and age-cohort-period (right) functions for alcohol treatment episodes per 100,000 people. 9](#_Toc172751873)

[Appendix G. Fit statistics for male-female interaction models. 10](#_Toc172751874)

[Appendix H. Sensitivity analyses results. 11](#_Toc172751875)

[Appendix H1. Excluding assessment-only episodes 11](#_Toc172751876)

[Appendix H2. Weighted least squares approach 11](#_Toc172751877)

[Appendix I. Fit statistics for age, period, and cohort models excluding assessment-only episodes. 12](#_Toc172751878)

[Appendix J. Plotted AIC (top) and BIC (bottom) values for age, period, and cohort models excluding assessment-only episodes. 13](#_Toc172751879)

[Appendix K. Weighted least squares APC Wald Tests for alcohol treatment episodes per 100,000 people. 14](#_Toc172751880)

[Appendix L. Estimated drifts with 95% confidence intervals from weighted least squares APC models for alcohol treatment episodes per 100,000 people. 15](#_Toc172751881)

[Appendix M. Estimated effects with 95% confidence intervals from weighted least squares APC models for alcohol treatment episodes per 100,000 people. 16](#_Toc172751882)

## Appendix A. STROBE Checklist.

|  | Item No | Recommendation | Page number |
| --- | --- | --- | --- |
| **Title and abstract** | 1 | (*a*) Indicate the study’s design with a commonly used term in the title or the abstract | 1 |
|  |  | (*b*) Provide in the abstract an informative and balanced summary of what was done and what was found | 2 |
| Introduction | | |  |
| Background/rationale | 2 | Explain the scientific background and rationale for the investigation being reported | 3-4 |
| Objectives | 3 | State specific objectives, including any prespecified hypotheses | 4 |
| Methods | | |  |
| Study design | 4 | Present key elements of study design early in the paper | 4-5 |
| Setting | 5 | Describe the setting, locations, and relevant dates, including periods of recruitment, exposure, follow-up, and data collection | 4-5 |
| Participants | 6 | (*a*) *Cohort study*—Give the eligibility criteria, and the sources and methods of selection of participants. Describe methods of follow-up  *Case-control study*—Give the eligibility criteria, and the sources and methods of case ascertainment and control selection. Give the rationale for the choice of cases and controls  *Cross-sectional study*—Give the eligibility criteria, and the sources and methods of selection of participants | 4-5 |
|  |  | (*b*) *Cohort study*—For matched studies, give matching criteria and number of exposed and unexposed  *Case-control study*—For matched studies, give matching criteria and the number of controls per case | N/A |
| Variables | 7 | Clearly define all outcomes, exposures, predictors, potential confounders, and effect modifiers. Give diagnostic criteria, if applicable | 4-5 |
| Data sources/ measurement | 8* | For each variable of interest, give sources of data and details of methods of assessment (measurement). Describe comparability of assessment methods if there is more than one group | 4-5 |
| Bias | 9 | Describe any efforts to address potential sources of bias | 5 |
| Study size | 10 | Explain how the study size was arrived at | 5 |
| Quantitative variables | 11 | Explain how quantitative variables were handled in the analyses. If applicable, describe which groupings were chosen and why | 5 |
| Statistical methods | 12 | (*a*) Describe all statistical methods, including those used to control for confounding | 5-7 |
|  |  | (*b*) Describe any methods used to examine subgroups and interactions | 6-7 |
|  |  | (*c*) Explain how missing data were addressed | 5 |
|  |  | (*d*) *Cohort study*—If applicable, explain how loss to follow-up was addressed  *Case-control study*—If applicable, explain how matching of cases and controls was addressed  *Cross-sectional study*—If applicable, describe analytical methods taking account of sampling strategy | 5 |
|  |  | (*e*) Describe any sensitivity analyses | 6-7 |

## Appendix B. Age-period and age-cohort plots of crude rates.

### Appendix B1. Age-specific alcohol treatment episodes per 100,000 population by period (top) and period-specific alcohol treatment episodes per 100,000 population by age group (bottom).


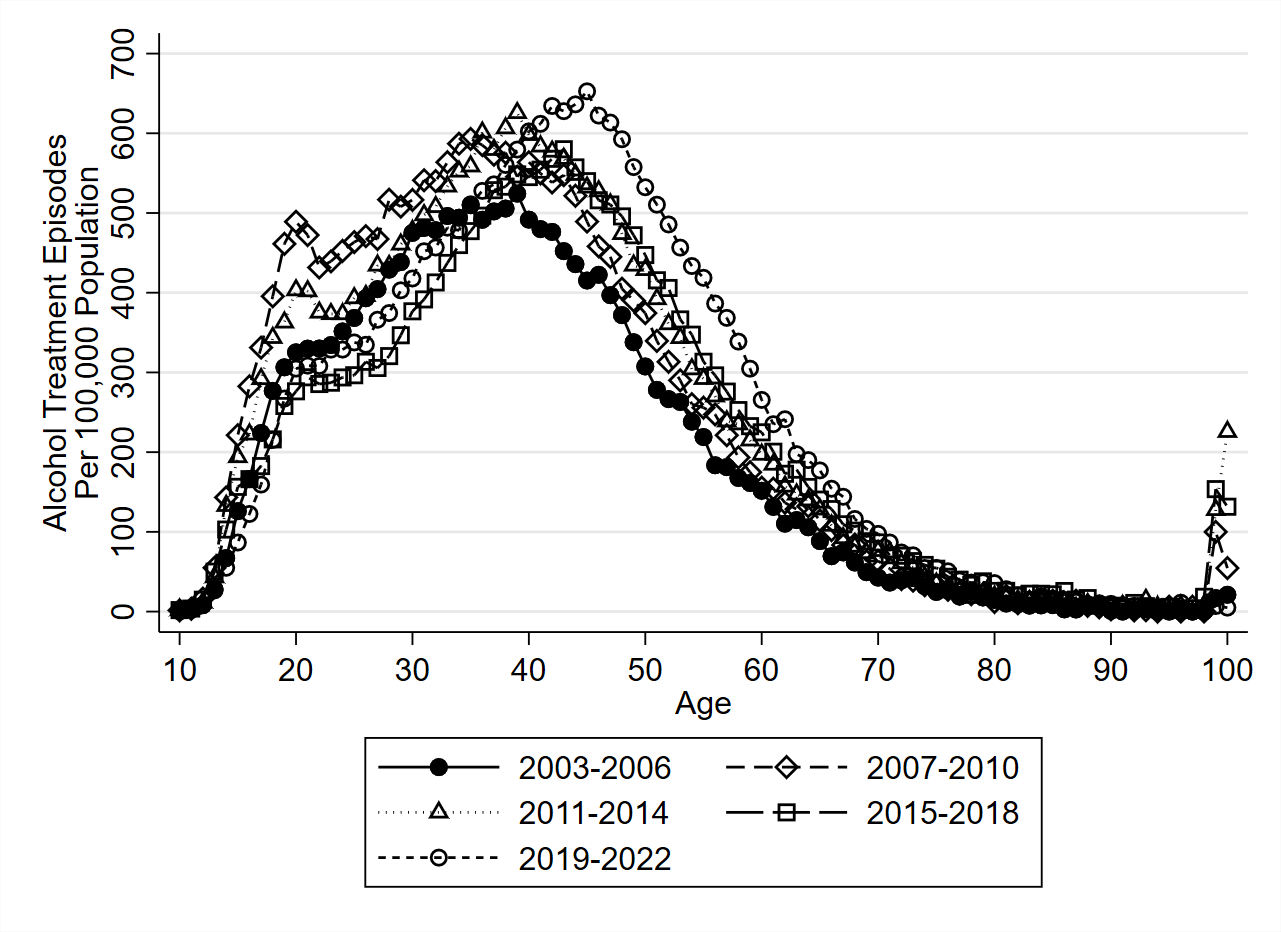


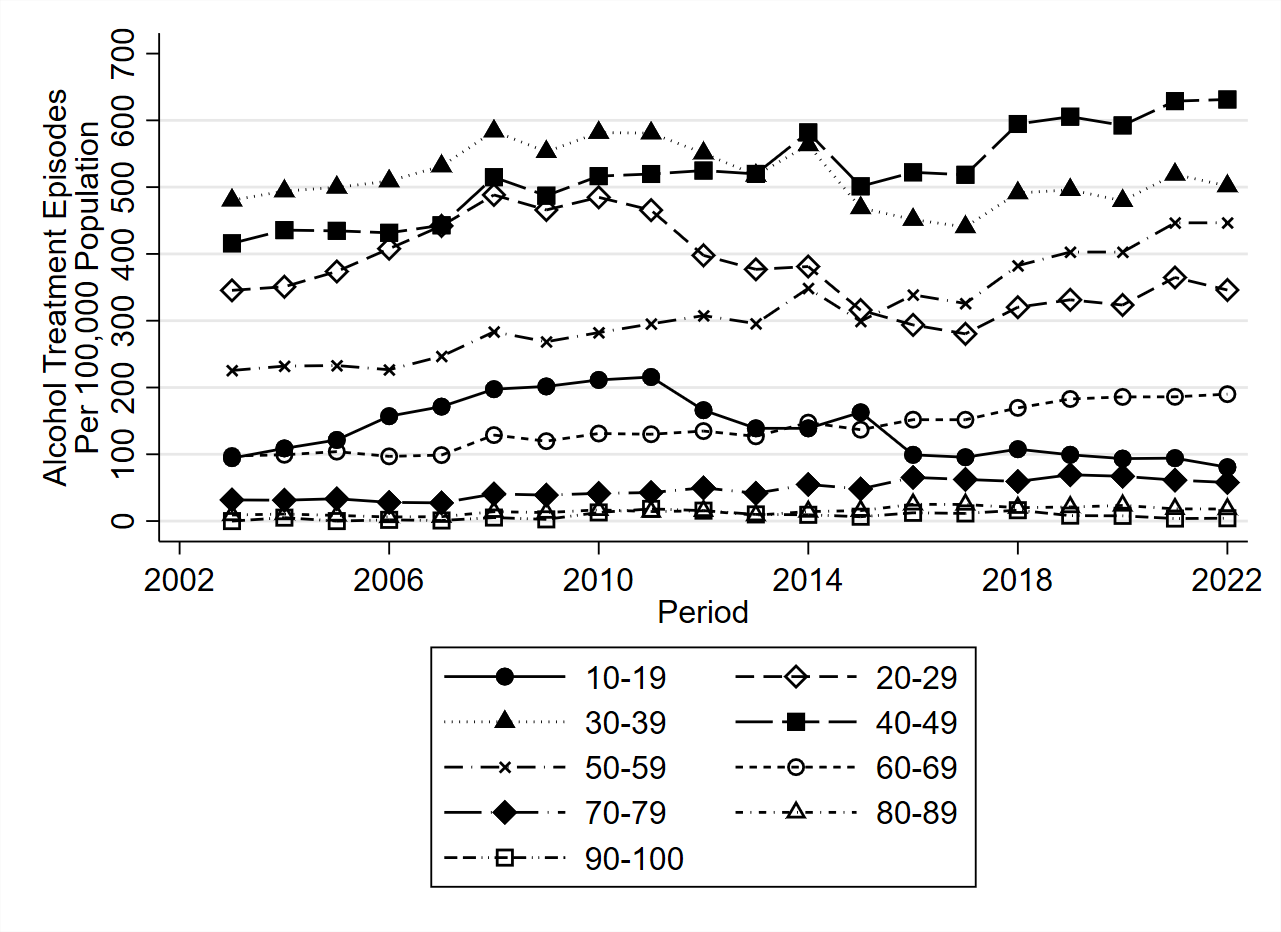


### Appendix B2. Age-specific alcohol treatment episodes per 100,000 population by birth cohort (top) and cohort-specific alcohol treatment episodes per 100,000 population by age group (bottom).


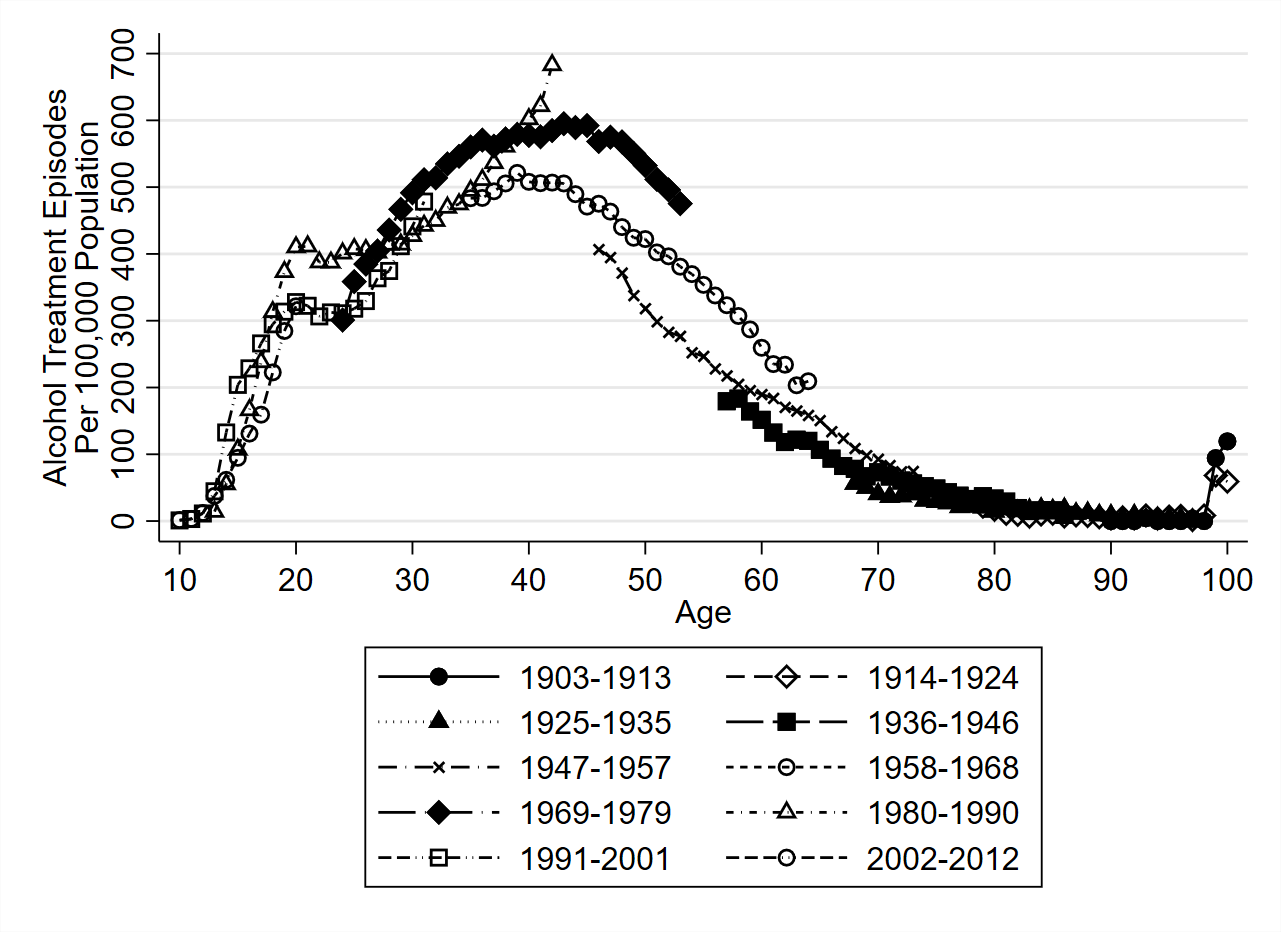

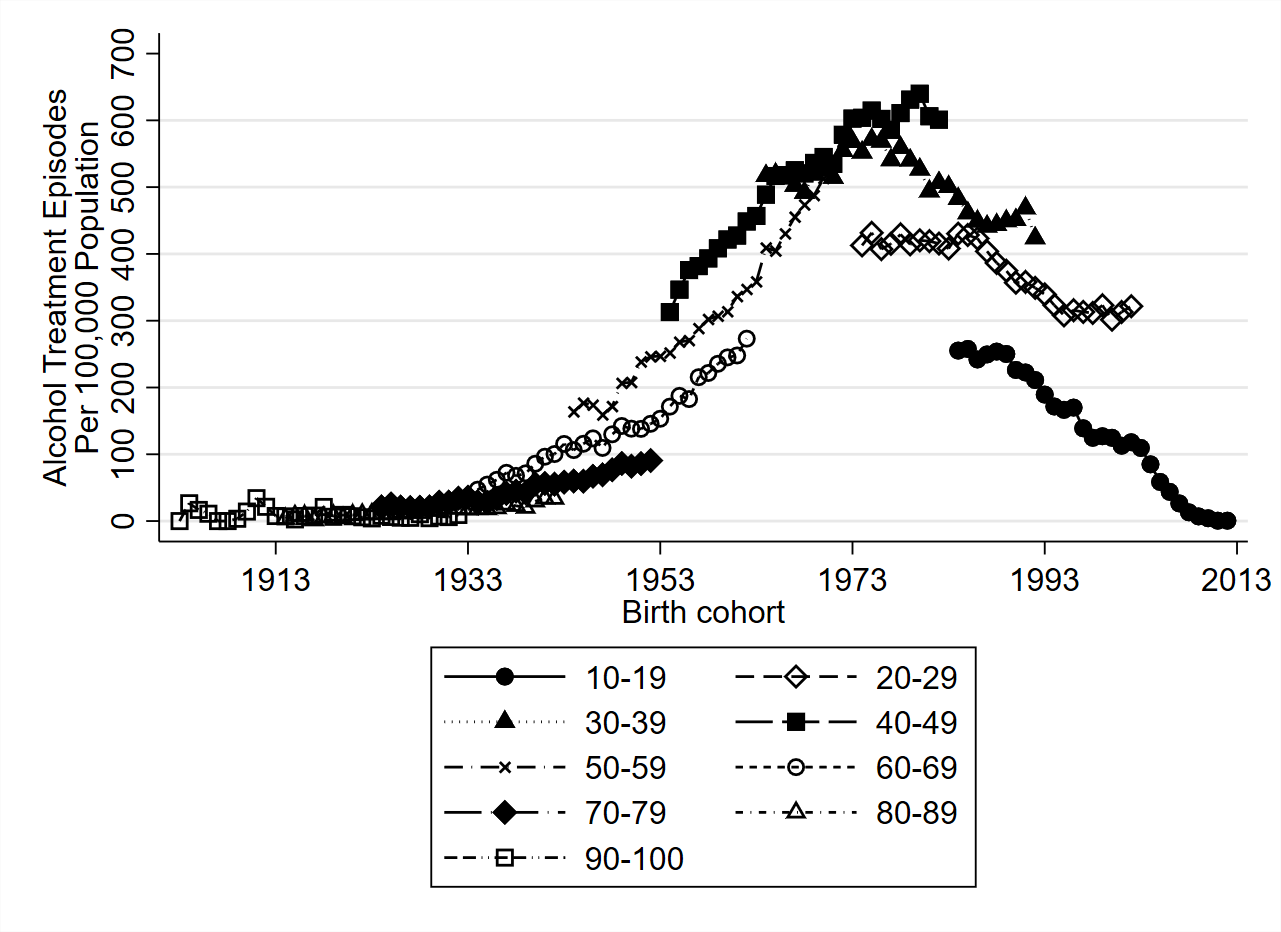


## Appendix C. Model selection details.

### Appendix C1. APC model

In selecting the APC model with the best fit, lower values for both AIC and BIC indicate a better model fit penalising for greater model complexity (i.e., more components or more cubic spline knots in this context). The motivation behind AIC is to find the best model for predictions whereas BIC attempts to find the best model to explain the data. BIC also generally penalises more complex models compared to AIC. To determine the number of equally spaced internal knots in the cubic spline functions of the final APC models, we compared AIC and BIC values for models with increasing knots for age, period, and cohort. We increased the knots for age, period, and cohort until AIC and BIC both showed no substantial improvement.

Appendix D shows the fit statistics for the full APC models, the age-cohort models, and the age-period models for different numbers of internal knots, and Appendix E shows the AIC and BIC values plotted at each internal knot count. Each of the AIC, BIC, and deviance statistics consistently showed that the full APC model had improved fit over the reduced models irrespective of the number of internal knots. All AC models had better fit than the AP models, indicating that cohort had a more pronounced effect than period irrespective of the number of internal knots.

Both AIC and BIC improved as the number of internal knots increased, although these criterion values generally showed less pronounced changes at knot counts higher than 11 (Appendix D and Appendix E). The model containing 11 internal knots for each of age, period, and cohort was thus chosen for our primary analyses.

### Appendix C2. Male-female APC interaction model

Due to the weighting method used for drift extraction in this APC modelling approach (31), a full interaction with all the APC terms may result in overfitting and skewed estimates, particularly when the male-female ratio of cases is uneven (Table 1). Therefore, we reduced the number of splines to model the interactions, decreasing the knots for age and cohort until AIC and BIC both showed no substantial improvement.

Though AIC was similar between the models, BIC showed that ten internal knots for each of age, period, and cohort had improved fit over other combinations with the reduced spline interaction, as well as improved fit over the full interaction model (Appendix G). Thus, for each of age, period, and cohort, ten equally spaced internal knots were used to describe non-linear effects in the reduced-spline male-female interaction models.

## Appendix D. Fit statistics for primary age, period, and cohort models.

| **Knots** | **Model** | **AIC** | **BIC** | **Log-likelihood** | **d.f.** | **Deviance** | **p** |
| --- | --- | --- | --- | --- | --- | --- | --- |
| 4 | APC | 38.80 | 44797.69 | -35288.65 | 1805 |  |  |
|  | AP | 49.76 | 64724.30 | -45266.97 | 1809 | 78303.73 | <.001 |
|  | AC | 41.40 | 49525.43 | -37667.53 | 1809 | 63104.86 | <.001 |
| 5 | APC | 36.19 | 40075.06 | -32916.07 | 1802 |  |  |
|  | AP | 47.08 | 59863.34 | -42828.98 | 1807 | 73427.75 | <.001 |
|  | AC | 38.94 | 45050.35 | -35422.48 | 1807 | 58614.77 | <.001 |
| 6 | APC | 31.14 | 30892.19 | -28313.38 | 1799 |  |  |
|  | AP | 42.07 | 50751.80 | -38265.70 | 1805 | 64301.20 | <.001 |
|  | AC | 33.94 | 35956.04 | -30867.82 | 1805 | 49505.44 | <.001 |
| 7 | APC | 26.28 | 22076.54 | -23894.29 | 1796 |  |  |
|  | AP | 37.24 | 41977.62 | -33871.10 | 1803 | 55512.01 | <.001 |
|  | AC | 29.03 | 27036.32 | -26400.45 | 1803 | 40570.70 | <.001 |
| 8 | APC | 22.72 | 15598.32 | -20643.92 | 1793 |  |  |
|  | AP | 33.76 | 35654.16 | -30701.87 | 1801 | 49173.53 | <.001 |
|  | AC | 25.60 | 20796.41 | -23272.99 | 1801 | 34315.78 | <.001 |
| 9 | APC | 19.74 | 10197.77 | -17932.39 | 1790 |  |  |
|  | AP | 30.75 | 30184.34 | -27959.45 | 1799 | 43688.69 | <.001 |
|  | AC | 22.75 | 15626.33 | -20680.45 | 1799 | 29130.69 | <.001 |
| 10 | APC | 17.39 | 5946.63 | -15795.56 | 1787 |  |  |
|  | AP | 28.32 | 25768.78 | -25744.16 | 1797 | 39258.13 | <.001 |
|  | AC | 20.31 | 11193.61 | -18456.58 | 1797 | 24682.96 | <.001 |
| **11** | **APC** | **15.81** | **3087.84** | **-14354.90** | **1784** |  |  |
|  | **AP** | **26.68** | **22809.58** | **-24257.06** | **1795** | **36283.91** | **<.001** |
|  | **AC** | **19.02** | **8859.89** | **-17282.21** | **1795** | **22334.23** | **<.001** |
| 12 | APC | 15.15 | 1889.50 | -13744.47 | 1781 |  |  |
|  | AP | 26.02 | 21607.21 | -23648.37 | 1793 | 35066.53 | <.001 |
|  | AC | 18.23 | 7426.21 | -16557.87 | 1793 | 20885.53 | <.001 |
| 13 | APC | 14.53 | 788.92 | -13182.92 | 1778 |  |  |
|  | AP | 25.39 | 20486.18 | -23080.35 | 1791 | 33930.49 | <.001 |
|  | AC | 17.88 | 6811.51 | -16243.01 | 1791 | 20255.82 | <.001 |
| 14 | APC | 14.51 | 760.16 | -13157.28 | 1775 |  |  |
|  | AP | 25.37 | 20450.65 | -23055.07 | 1789 | 33879.94 | <.001 |
|  | AC | 17.83 | 6727.57 | -16193.53 | 1789 | 20156.86 | <.001 |
| 15 | APC | 14.16 | 135.29 | -12833.59 | 1772 |  |  |
|  | AP | 25.06 | 19902.63 | -22773.55 | 1787 | 33316.91 | <.001 |
|  | AC | 17.52 | 6169.67 | -15907.07 | 1787 | 19583.94 | <.001 |
| 16 | APC | 14.10 | 56.63 | -12782.99 | 1769 |  |  |
|  | AP | 24.99 | 19787.74 | -22708.61 | 1785 | 33187.01 | <.001 |
|  | AC | 17.48 | 6122.40 | -15875.93 | 1785 | 19521.66 | <.001 |
| 17 | APC | 13.98 | -142.42 | -12672.21 | 1766 |  |  |
|  | AP | 24.89 | 19606.55 | -22610.51 | 1783 | 32990.81 | <.001 |
|  | AC | 17.48 | 6119.86 | -15867.16 | 1783 | 19504.12 | <.001 |
| 18 | APC | 13.67 | -693.48 | -12385.42 | 1763 |  |  |
|  | AP | 24.62 | 19132.40 | -22365.92 | 1781 | 32501.64 | <.001 |
|  | AC | 17.23 | 5677.18 | -15638.31 | 1781 | 19046.42 | <.001 |

Note. Knots = number of equally spaced internal knots for age, period, and cohort; APC = age-period-cohort; AP = age-period; AC = age-cohort; AIC = Akaike Information Criterion, smaller values indicate better model fit; BIC = Bayesian Information Criterion, smaller values indicate better model fit; d.f. = degrees of freedom. Bolded rows indicate chosen model.

## Appendix E. Plotted AIC (top) and BIC (bottom) values for primary age, period, and cohort models.

## Appendix F. Residuals from APC models using age-period-cohort (left) and age-cohort-period (right) functions for alcohol treatment episodes per 100,000 people.


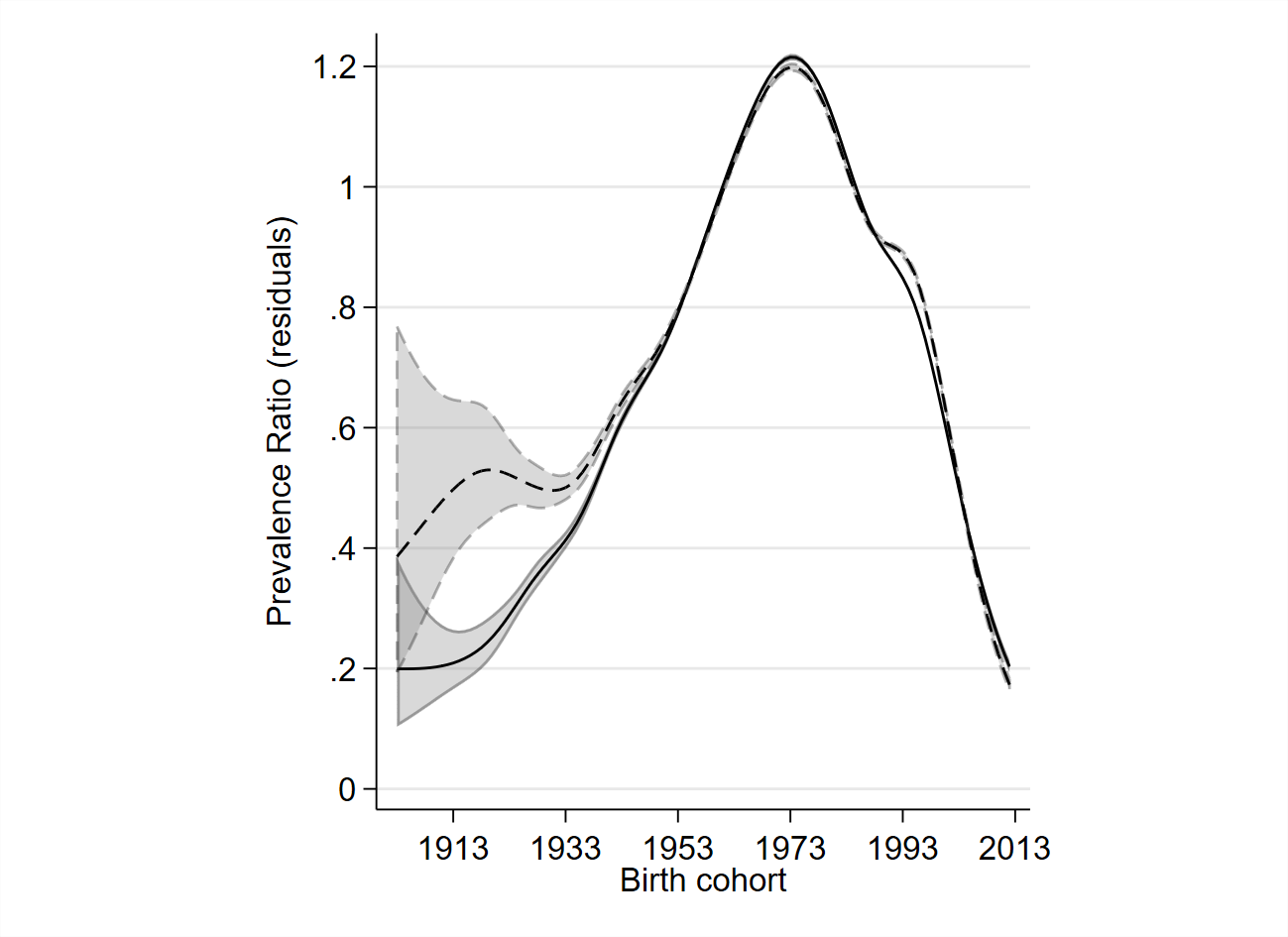

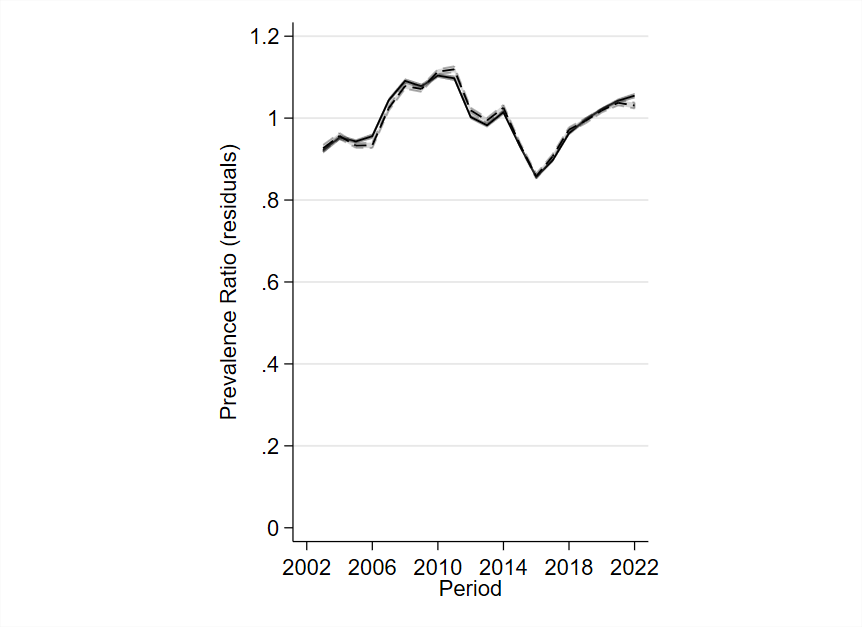


Note. Using 11 knots for each of age, period, and cohort. Solid lines are residuals from the primary models, dashed lines are residuals from the sensitivity models where assessment-only treatment episodes are excluded.

## Appendix G. Fit statistics for male-female interaction models.

| **Model** | **Knots** | **AIC** | **BIC** | **Log-likelihood** | **d.f.** |
| --- | --- | --- | --- | --- | --- |
| Full interaction | 11 | 12.31 | -6437.04 | -22343.74 | 3580 |
| Reduced Splines | 10 | 11.96 | -7724.86 | -21703.93 | 3581 |
|  | 9 | 12.05 | -7393.66 | -21877.73 | 3583 |
|  | 8 | 12.08 | -7289.33 | -21938.09 | 3585 |
|  | 7 | 12.08 | -7315.72 | -21933.10 | 3587 |
|  | 6 | 12.13 | -7144.09 | -22027.11 | 3589 |

Note. Knots = number of equally spaced internal knots for age and cohort; AIC = Akaike Information Criterion, smaller values indicate better model fit; BIC = Bayesian Information Criterion, smaller values indicate better model fit; d.f. = degrees of freedom. Bolded row indicates chosen model.

## Appendix H. Sensitivity analyses results.

### Appendix H1. Excluding assessment-only episodes

Rates of alcohol treatment episodes excluding those that were categorised as assessment-only are shown in Table 1 of the main manuscript. Fit statistics showed the same pattern as that of the primary analyses, with a more pronounced cohort effect than period and improved fit for the full APC models over the AC and AP models at all knot counts (Appendix I). We chose 11 internal knots for each of age, period, and cohort primarily for comparison with the primary analyses, though these 11-knot models also had great balance of model fit and interpretability (Appendix J). Cross-sectional (AP-C; manuscript Figure 2 top left dashed line) and longitudinal (AC-P; Figure 2 bottom left dashed line) age effects showed similar trends to the primary analyses, with a larger difference in primary vs sensitivity effect size between the longitudinal age effects than the cross-sectional age effects. Period effects were almost identical to that of the primary analysis (Figure 2 top right dashed line) except for the PR from 2018 onwards showing a less steep increase. Cohort effects were likewise broadly similar to the primary analysis (Figure 2 bottom right dashed line), however the difference in PRs was more pronounced in the sensitivity model from the 1903 to 1920s birth cohorts.

### Appendix H2. Weighted least squares approach

Wald tests for net drift and local drifts using the WLS approach were significant, indicating that fitted temporal trends in alcohol treatment episode rates varied over time and that temporal patterns varied by age, respectively (Appendix K). Net drift in alcohol treatment episode rates was 2.48 (95% CI = 1.97, 2.99). Local drifts showed that the percentage change in alcohol treatment episode rates decreased over time from age 10 to 35 years, with generally a larger decrease with younger age (Appendix L). The percentage change in alcohol treatment episode rates increased over time from aged 37 years, with an increasingly larger increase up to age 55 years and similar annual percentage change between ages 56 to 85 years. Estimates for age 87 onwards were less precise, and it was unclear whether there was any increase or decrease in annual percentage change at these ages.

Age, period, and cohort effects were also significant (Appendix K). Cross-sectional age effects differed somewhat from the primary analyses, with a lifetime peak in alcohol treatment episode rates at age 20 years (456.51 [411.45-506.51]; Appendix M, top left) rather than 37 years. However, the cross-sectional age trend resembled that of the primary analyses, with increasing rates up to the early 20s, dip at age 26 years, stable between ages 35 to 45 years, and decline thereafter until the mid-late 90s. Longitudinal age effects were very similar to the primary analyses (Appendix M, bottom left). Period effects were also similar, with the only substantial difference being that the PR was stable at around 1.00 between 2015 and 2017 in the WLS model (Appendix M, top right), whereas the primary analyses showed a dip in 2016. Cohort effects were likewise similar to the primary analyses, peaking around 1974 to 1979 and decreasing the further a birth cohort was from these peak cohorts (Appendix M, bottom right).

## Appendix I. Fit statistics for age, period, and cohort models excluding assessment-only episodes.

| **Knots** | **Model** | **AIC** | **BIC** | **Log-likelihood** | **d.f.** | **Deviance** | **p** |
| --- | --- | --- | --- | --- | --- | --- | --- |
| 4 | APC | 34.56 | 37519.44 | -31432.98 | 1805 | 51068.84 |  |
|  | AP | 42.03 | 51100.42 | -38238.48 | 1809 | 64679.84 | <.001 |
|  | AC | 36.67 | 41339.76 | -33358.15 | 1809 | 54919.18 | <.001 |
| 5 | APC | 32.79 | 34319.89 | -29821.94 | 1802 | 47846.77 |  |
|  | AP | 40.21 | 47791.48 | -36576.50 | 1807 | 61355.89 | <.001 |
|  | AC | 35.02 | 38357.58 | -31859.55 | 1807 | 51922.00 | <.001 |
| 6 | APC | 28.79 | 27055.74 | -26178.61 | 1799 | 40560.10 |  |
|  | AP | 36.32 | 40717.72 | -33032.12 | 1805 | 54267.12 | <.001 |
|  | AC | 31.02 | 31078.97 | -28212.74 | 1805 | 44628.37 | <.001 |
| 7 | APC | 24.61 | 19458.74 | -22368.85 | 1796 | 32940.58 |  |
|  | AP | 32.31 | 33432.19 | -29381.85 | 1803 | 46966.58 | <.001 |
|  | AC | 26.79 | 23392.94 | -24362.22 | 1803 | 36927.33 | <.001 |
| 8 | APC | 21.30 | 13464.30 | -19360.37 | 1793 | 26923.62 |  |
|  | AP | 29.21 | 27807.49 | -26561.99 | 1801 | 41326.86 | <.001 |
|  | AC | 23.60 | 17604.55 | -21460.52 | 1801 | 31123.92 | <.001 |
| 9 | APC | 18.48 | 8330.68 | -16782.29 | 1790 | 21767.48 |  |
|  | AP | 26.42 | 22735.76 | -24018.62 | 1799 | 36240.12 | <.001 |
|  | AC | 20.88 | 12652.84 | -18977.15 | 1799 | 26157.19 | <.001 |
| 10 | APC | 16.13 | 4072.49 | -14641.94 | 1787 | 17486.77 |  |
|  | AP | 24.01 | 18362.83 | -21824.64 | 1797 | 31852.18 | <.001 |
|  | AC | 18.45 | 8245.60 | -16766.03 | 1797 | 21734.94 | <.001 |
| **11** | **APC** | **14.49** | **1110.10** | **-13149.49** | **1784** | **14501.86** |  |
|  | **AP** | **22.32** | **15292.13** | **-20281.79** | **1795** | **28766.47** | **<.001** |
|  | **AC** | **17.12** | **5838.72** | **-15555.08** | **1795** | **19313.05** | **<.001** |
| 12 | APC | 13.76 | -204.16 | -12481.09 | 1781 | 13165.08 |  |
|  | AP | 21.58 | 13962.10 | -19609.27 | 1793 | 27421.42 | <.001 |
|  | AC | 16.26 | 4279.17 | -14767.80 | 1793 | 17738.49 | <.001 |
| 13 | APC | 13.03 | -1504.65 | -11819.59 | 1778 | 11842.07 |  |
|  | AP | 20.85 | 12646.43 | -18943.92 | 1791 | 26090.74 | <.001 |
|  | AC | 15.85 | 3556.50 | -14398.96 | 1791 | 17000.80 | <.001 |
| 14 | APC | 12.98 | -1593.54 | -11763.89 | 1775 | 11730.66 |  |
|  | AP | 20.79 | 12542.91 | -18884.66 | 1789 | 25972.20 | <.001 |
|  | AC | 15.79 | 3442.27 | -14334.34 | 1789 | 16871.57 | <.001 |
| 15 | APC | 12.62 | -2218.31 | -11440.24 | 1772 | 11083.37 |  |
|  | AP | 20.46 | 11967.44 | -18589.41 | 1787 | 25381.72 | <.001 |
|  | AC | 15.46 | 2851.08 | -14031.24 | 1787 | 16265.36 | <.001 |
| 16 | APC | 12.54 | -2354.91 | -11360.68 | 1769 | 10924.25 |  |
|  | AP | 20.37 | 11799.33 | -18497.85 | 1785 | 25198.60 | <.001 |
|  | AC | 15.40 | 2766.45 | -13981.42 | 1785 | 16165.72 | <.001 |
| 17 | APC | 12.42 | -2548.59 | -11252.58 | 1766 | 10708.06 |  |
|  | AP | 20.26 | 11617.72 | -18399.55 | 1783 | 25001.98 | <.001 |
|  | AC | 15.40 | 2767.86 | -13974.61 | 1783 | 16152.11 | <.001 |
| 18 | APC | 12.10 | -3119.98 | -10955.63 | 1763 | 10114.14 |  |
|  | AP | 19.97 | 11099.67 | -18133.01 | 1781 | 24468.91 | <.001 |
|  | AC | 15.16 | 2355.47 | -13760.91 | 1781 | 15724.71 | <.001 |

Note. Knots = number of equally spaced internal knots for age, period, and cohort; APC = age-period-cohort; AP = age-period; AC = age-cohort; AIC = Akaike Information Criterion, smaller values indicate better model fit; BIC = Bayesian Information Criterion, smaller values indicate better model fit; d.f. = degrees of freedom. Bolded rows indicate chosen model.

## Appendix J. Plotted AIC (top) and BIC (bottom) values for age, period, and cohort models excluding assessment-only episodes.

## Appendix K. Weighted least squares APC Wald Tests for alcohol treatment episodes per 100,000 people.

| **Null hypothesis** | **X^2^** | **d.f.** | **P-value** |
| --- | --- | --- | --- |
| **Net drift = 0** | 93.05 | 1 | <.001 |
| **Local drifts = net drift** | 2995.95 | 90 | <.001 |
| **All age deviations = 0** | 6680.52 | 89 | <.001 |
| **All period deviations = 0** | 938.98 | 18 | <.001 |
| **All cohort deviations = 0** | 3031.24 | 108 | <.001 |
| **All period PRs = 1** | 1030.50 | 19 | <.001 |
| **All cohort PRs = 1** | 3436.49 | 109 | <.001 |

Note. d.f. = degrees of freedom. PR = prevalence ratio. P < .05 indicates that there is evidence against the null hypothesis. Net drift is the age-standardised annual percentage change in alcohol treatment episode rates. Local drifts are the age-specific annual percentage change. The null hypothesis for net drift refers to constant temporal trends and the null hypothesis for local drifts refers to constant temporal trends across ages. Null hypotheses for age, period, and cohort deviations are referring to fitted trends for the corresponding component being log-linear. Null hypotheses for period and cohort RRs are referring to constant age curves across period and cohort, respectively.

## Appendix L. Estimated drifts with 95% confidence intervals from weighted least squares APC models for alcohol treatment episodes per 100,000 people.


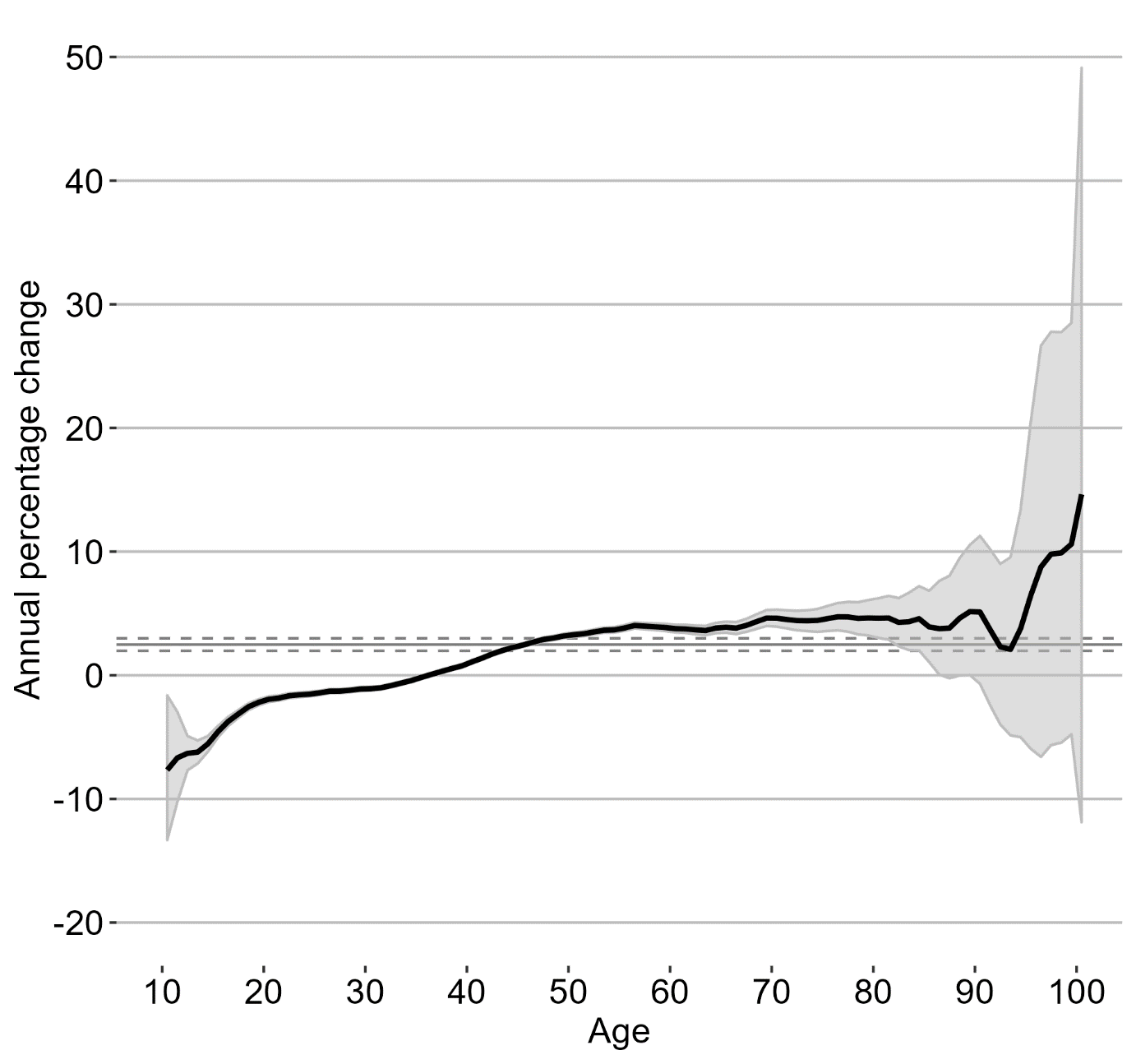


Note. Net drift (annual percentage change in expected age-adjusted rates) is represented by the dark grey horizontal line with 95% confidence intervals shown as dashed lines. Local drifts (annual change in expected age-specific rates) are represented by the black line with 95% confidence intervals shown as the shaded grey area.

## Appendix M. Estimated effects with 95% confidence intervals from weighted least squares APC models for alcohol treatment episodes per 100,000 people.


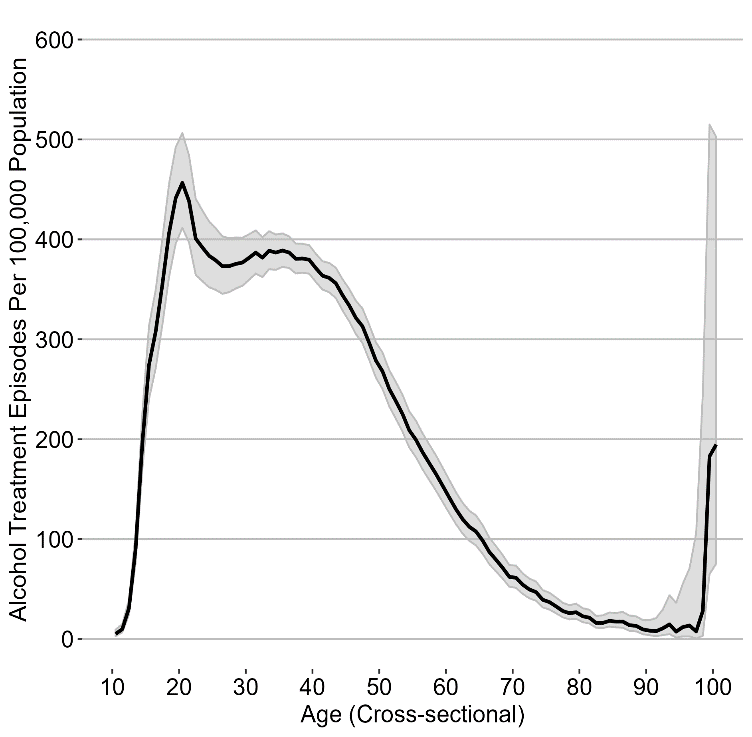

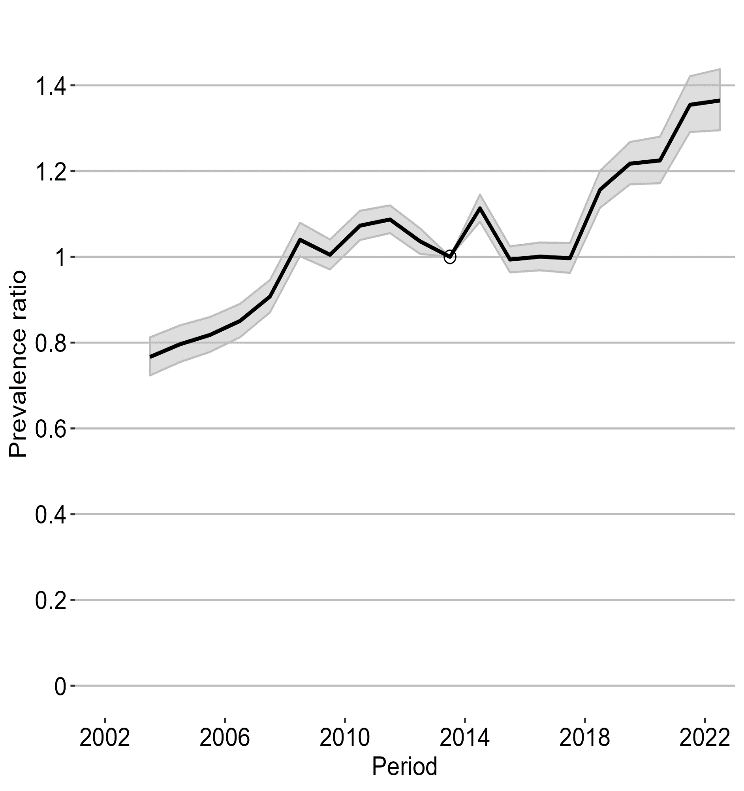


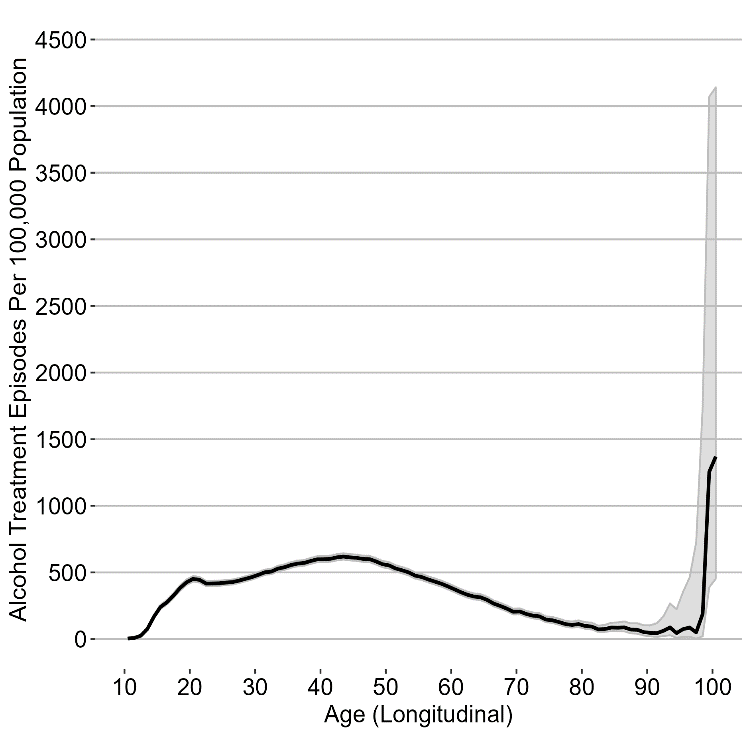

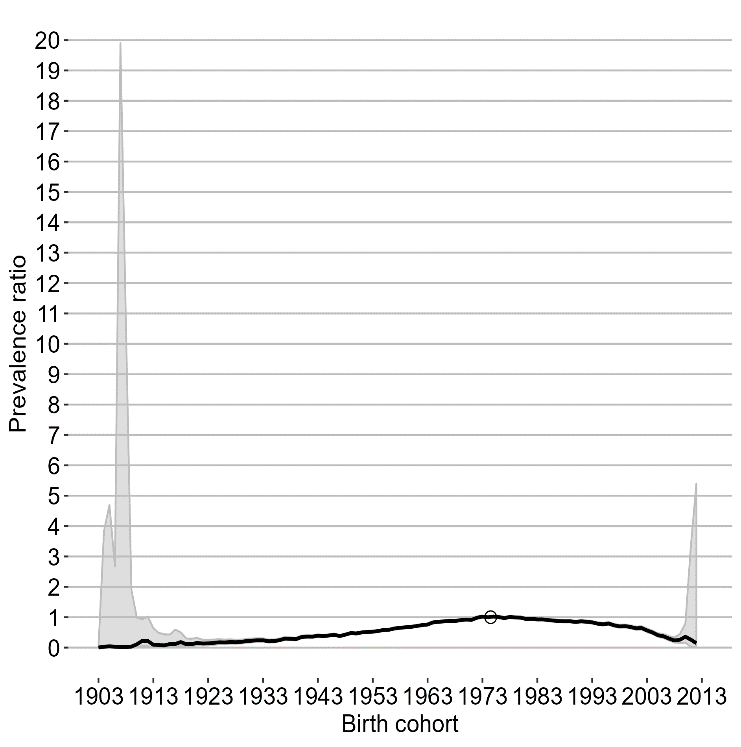


Note. Cross-sectional age effects for the reference period of 2013 (circle) are shown on the top left and period effects are shown on the top right. Longitudinal age effects for the reference birth cohort of 1974 (circle) are shown on the bottom left and cohort effects are shown in the bottom right.
